# Quantifying Disorder through Conditional Entropy: An Application to Fluid Mixing

**DOI:** 10.1371/journal.pone.0065617

**Published:** 2013-06-10

**Authors:** Giovanni B. Brandani, Marieke Schor, Cait E. MacPhee, Helmut Grubmüller, Ulrich Zachariae, Davide Marenduzzo

**Affiliations:** 1 SUPA, School of Physics and Astronomy, University of Edinburgh, Edinburgh, Scotland, United Kingdom; 2 Department of Theoretical and Computational Biophysics, Max Planck Institute for Biophysical Chemistry, Göttingen, Germany; 3 Division of Computational Biology, College of Life Sciences, University of Dundee, Dundee, United Kingdom; 4 Division of Physics, School of Engineering, Physics and Mathematics, University of Dundee, Nethergate, Dundee, United Kingdom; University of Zurich, Switzerland

## Abstract

In this paper, we present a method to quantify the extent of disorder in a system by using conditional entropies. Our approach is especially useful when other global, or mean field, measures of disorder fail. The method is equally suited for both continuum and lattice models, and it can be made rigorous for the latter. We apply it to mixing and demixing in multicomponent fluid membranes, and show that it has advantages over previous measures based on Shannon entropies, such as a much diminished dependence on binning and the ability to capture local correlations. Further potential applications are very diverse, and could include the study of local and global order in fluid mixtures, liquid crystals, magnetic materials, and particularly biomolecular systems.

## Introduction

### Disorder-order Transitions and the Shannon Entropy

Disorder-order transitions are important physical phenomena that are commonly addressed both by simulations and experiments. They play a major role in the description of the behaviour of liquids and solids [1], the level of spin alignment in ferromagnetic systems [2], and domain formation in biological fluids such as membranes [3]. Because of the widespread occurrence of these phenomena, it is desirable to obtain methods to quantify the local and global level of disorder in a system, which can be generally applied to a broad range of systems.

At equilibrium, disorder can be quantified by the thermodynamic entropy, which typically necessitates the explicit knowledge of the partition function of the system [4]. However, since experiments or simulations are often monitoring systems away from equilibrium, and the system Hamiltonian is often unknown in experiments, a simple formulation using the thermodynamic entropy is not readily available. In order to develop a useful, more general measure of system disorder, it is therefore necessary to consider alternative approaches. We here use the Shannon entropy [5] from information theory, which is defined as.
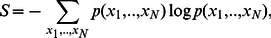
(1)where the sum is performed over all possible configurations of the system, and 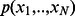 represents the frequency of occurrence of the N-particle state 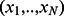. The information-theoretical derivation of entropy is not restricted to thermodynamic equilibrium and it can be computed directly from the observed frequencies of configurations. Hence, it can be a useful tool to describe any macro-state of the system. In previous work, the Shannon entropy has been used successfully to quantify the order in fluid mixtures [6].

Here we present a widely applicable method to quantify disorder in systems at or away from equilibrium, based on Shannon and conditional entropy. It can be easily implemented for use on physical simulation and experimental datasets. Our approach employs the concept of conditional entropy, which derives from the measure of entropy in images [7] and complex networks [8]. Its main advantage is the capturing of local correlations between particles or states, which are an important factor for characterising disorder-order transitions.

The simplest estimate of the Shannon entropy is given by the mean field approximation.
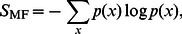
(2)where 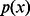 is the probability of the single particle state. This is a drastic simplification, which is only accurate when the 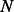-point joint probability distribution can be factorised into the single particle probabilities 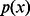, i.e. when there are no correlations between different particles. For example, this is a good approximation for the Ising ferromagnet in the high temperature regime, when the correlations between neighbouring spins (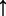 or 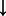) are small. However, it highly overestimates the value of the entropy close to the critical temperature, as the correlation length diverges to infinity at continuous phase transitions [4]. Since the entropy is also defined as the volume of phase space available to the system, correlations always lead to a decrease of entropy as they are equivalent to constraints on the configurations of the system which reduce the volume of the phase space.

After describing the problem of quantifying disorder in multicomponent systems, we introduce our new method based on the conditional entropy, and we validate it by computing the entropy in the Ising model. We then focus on fluid mixing as a case study which best illustrates our approach and which is an important and well studied problem in the biophysics of lipid membranes. Its implications range from cellular organisation [9] and endocytotic processes [10] to lipid raft formation [3], [11]. Our method captures local correlations, which makes it especially useful to quantify the extent of disorder in systems that typically form small domains on a local level which leave little or no signal in mean field or global indicators, such as in the case of lipid mixing/demixing.

### The Entropy of Mixing

The entropy of mixing is defined as the increase of disorder in a multi-component system upon transition from a fully demixed (partitioned) to an ideally mixed state [12]. Per particle, the entropy of mixing is defined between zero, in the fully demixed case, and the maximum value of.
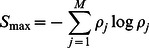
(3)in the fully mixed case, where 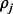 is the mole fraction of component 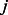 and 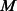 is the total number of components in the system. If we assume our system to be distributed on a lattice, the entropy of mixing is a measure of the uncertainty of the component type at each lattice site. Note that we omit the constant 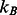 in the expression of the entropy in order to be consistent with the Shannon formulation.

Evaluating the disorder of composite systems is not trivial. The mean field approximation is bound to lead to the maximal entropy, as in Eq. (2) the frequency of each component type is equated with its mole fraction, such that a formulation for states of the system between full mixing and demixing is not readily available. In Ref. [6], Camesasca et al. have developed an approach to evaluate the entropy of mixing which overcomes the issue of the trivial mean field result. Their main idea was to subdivide the system into regions, and then evaluate the Shannon entropy of each region 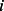 using 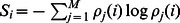, where 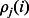 is the number of particles of type 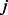 inside 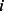 divided by the total number of particles in 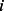. The entropy of mixing of the whole system is then estimated as the average of the entropies over all subregions.
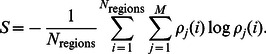
(4)


This quantity has the correct limiting values of zero and 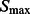, and is able to provide a good measure of the level of mixing of the different components. In Ref. [13], a continuous version of Eq. (4) has been successfully applied to multi-component lipid membranes.

However, as noted by the authors [6], this estimate shows high fluctuations dependent on the choice of the number of subregions. Hence, the use of Eq. (4) becomes especially problematic when the local organisation of the system at the inter-particle length-scale is of interest. Our goal was, therefore, to develop an approach with a broader generality and, in particular, applicable to systems with strong correlations between neighbouring regions, for instance to describe domain formation in lipid membranes.

## Results and Discussion

### A New Quantification Based on the Conditional Entropy

To enable both a rigorous derivation of our method and a comparison with other approaches, we start from lattice models, which are employed to simulate many of our systems of interest, including fluid mixtures [14] and lipid membranes [15], [16]. We consider a translationally invariant system composed of 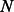 identical particles on a lattice, with 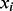 as the possible states, e.g. occupancies by a system component, of lattice site 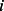. To account for correlations between sites, we must go beyond the mean-field approximation and abandon the assumption that the full probability distribution can be factorised into single particle probabilities.

In a one-dimensional lattice, the Bethe approximation [17] assumes that the 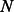-point probability can be written in terms of 1- and 2-point probabilities. The entropy per site thus takes the form.
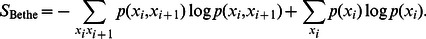
(5)


The above equation is exact if only nearest neighbour interactions are present [18]. An improvement to the Bethe approximation for studying lattice systems of any dimension is the Kikuchi approximation [19], [20], also known as cluster variation method. On a square two-dimensional lattice, the entropy per lattice site can be expressed as a sum of the entropies of three basic clusters of the lattice.
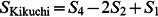
(6)with 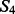 being the entropy of four neighbouring lattice sites arranged in a square, 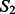 the entropy of two nearest neighbours, and 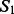 the single-particle entropy. The cluster variation method has been extremely successful for the theoretical description of lattice models [21], but its correct implementation depends on the specific network of interactions in the system.

To provide a more straightforward estimate of the entropy from computational or experimental data, where an underlying network structure may not be present, we follow a different direction, and start from the concepts developed by Shannon in Ref. [5]. There, for every realisation of a certain variable x, the conditional probability for another variable y to occur simultaneously is defined as 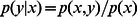, where 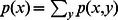. The conditional entropy 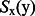 is defined as the average entropy of y for each value of x weighted by the probability of obtaining that particular value of 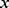:
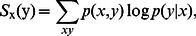
(7)


so that the entropy of two variables x and y can be written as 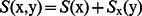. Generalising this equation to the entropy of a 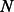-particle system gives:
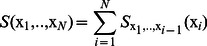
(8)


The correlations relevant to the calculation of the entropy are usually confined within a small neighbourhood of each particle [21]. If we use this approximation together with the translational invariance of the system, we obtain:
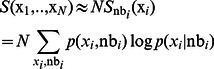
(9)where 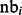 represents the state of the particles in the neighbourhood of 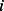, confined within a certain cut-off distance from 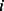. This result can be made rigorous on a lattice provided that we take into account the double counting of the sites in the neighbourhood.

Eq. (9) states that the Shannon entropy per particle can be approximated as the conditional entropy of each particle with respect to a variable representing the state of its neighbourhood. In the following, we will employ Eq. (9) as a measure of disorder in multi-component systems.

We note that the Bethe and Kikuchi approximations can be regarded as special cases of Eq. (9). In particular, by defining the neighbourhood as the single nearest neighbour, we obtain Eq. (5) for one-dimensional systems, whereas by defining it as the state of the three neighbours forming a square with 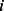, we obtain Eq. (6) for the lattice in two dimensions.

Moreover, Shannon proved in Ref. [5] that the entropy per symbol of an information source is equal to the limit for 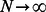 of the conditional entropy of one symbol with respect to the preceding sequence of 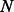 symbols,
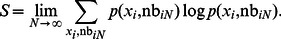
(10)


This theorem states that the approximation of the entropy given in Eq. (9) is rigorous in one dimension, and its generalisation suggests that the accuracy of the estimate increases with the size of the chosen neighbourhood. In the following, we will test and apply Eq. (9) on disorder-order transitions in lattice and continuous model systems.

### Test on the Ferromagnetic Ising Model in 2d

A useful example to test our approximation is the ferromagnetic Ising model on a two-dimensional square lattice [4], since it shows a continuous order-disorder transition at temperature 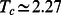, and the entropy 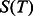 has been derived analytically by Onsager [2]. The Hamiltonian is 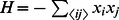, where the spins can take values 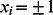.

We performed a Monte-Carlo simulation of this Ising model and calculated the entropy from equilibrium ensembles using different approximations: mean field, Kikuchi and conditional entropy. We discuss both the Glauber dynamics (system GD), which refers to the standard Ising model in Ref. [2], and the Kawasaki dynamics (system KD), where the magnetisation is fixed to zero, and it can be considered as a model for a binary mixture. In the former case we will compare our approximations to the exact solution.

**Figure 1:**
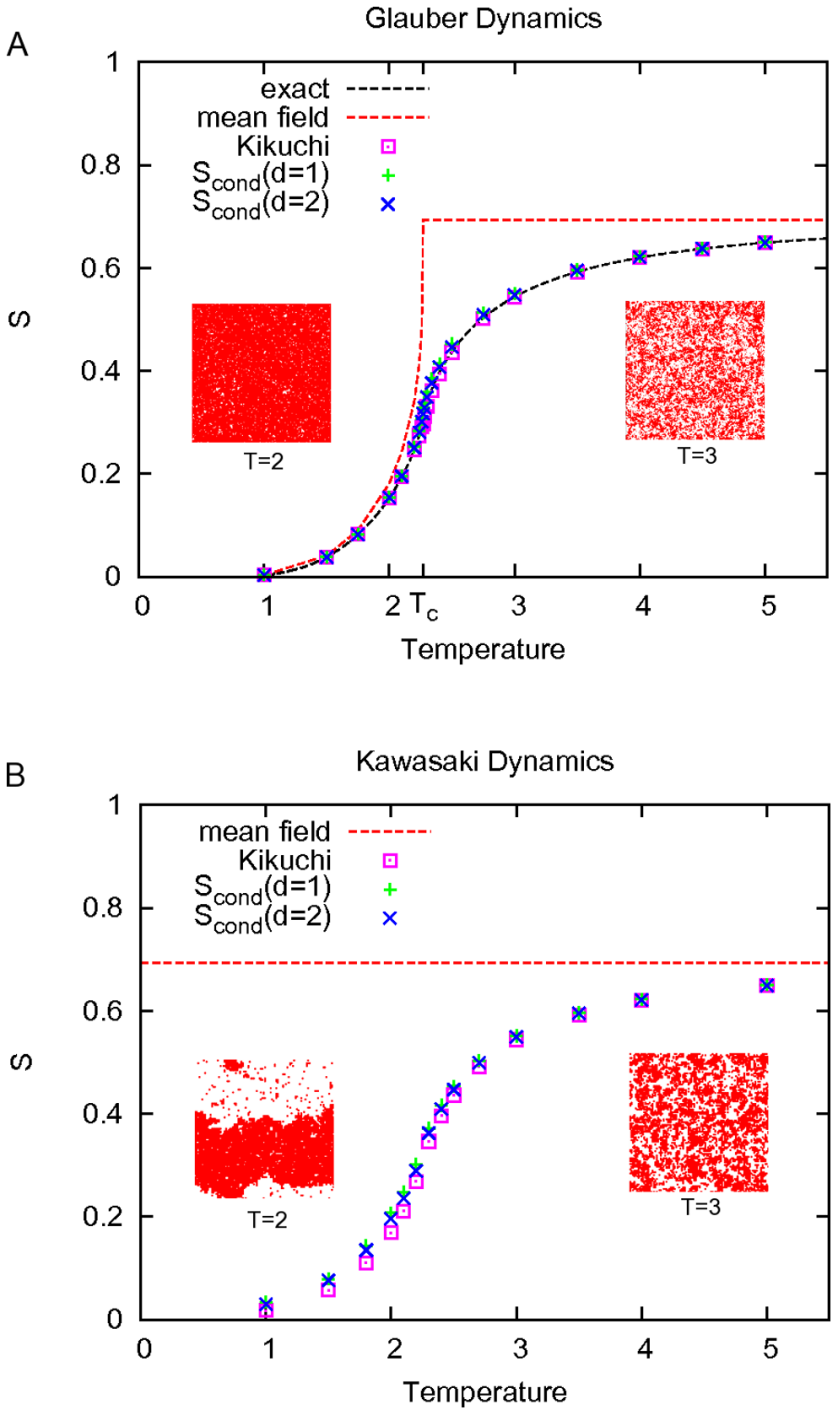
Entropy of the Ising model. Entropy per particle 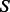 for the Ising model on a square lattice as a function of the temperature 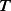. (A) Glauber Dynamics (200×200 lattice). (B) Kawasaki dynamics with fixed zero magnetisation (100×100 lattice). We estimated 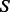 from equilibrium ensembles of Monte-Carlo simulations using different approximations: mean field, Kikuchi and conditional entropy. In (A) we also compare our results with the exact solution obtained by Onsager [2]. The neighbourhood in 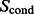 is defined as the set of lattice sites within a maximum distance 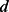 and in the upper half-plane from each site.

In the mean field approximation, the entropy is given by Eq. (2). Because the system is invariant under translations, we can estimate 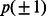 as the ensemble average of the number of sites with spin 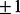 in each configuration divided by 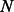. If the average magnetisation is zero, this approximation always gives 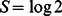, which is exact only in the limit 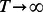. However, as it ignores correlations, it highly overestimates the entropy of GD as we move towards the transition temperature (Fig. 1A ). By contrast, the Kikuchi approximation, given by Eq. (6), provides an excellent measure of the entropy of the Ising model (Fig. 1A).

Our formulation in terms of the conditional entropy (Eq. (9)) requires knowledge of the frequency with which states occur in the neighbourhood of each lattice site. This leads to the question how the neighbourhood and its states should be defined. This choice is crucial for the accuracy of our approach, since we assume that the correlations relevant to the calculation of the entropy are confined within the neighbourhood. As suggested by the Kikuchi approximation, the inclusion of nearest and next-nearest neighbours is sufficient to obtain a good estimate for the entropy.

In the case described here, 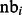 is associated with the spins 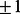 of the neighbourhood. The neighbourhood includes the sites confined within a maximum distance 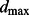, and located in the upper half-plane from each site. Taking into account only half of the neighbour sites is necessary to avoid double-counting of the interactions, and to make the approximation of the Shannon entropy rigorous. In Fig. 1, we show two possible choices of cut-off distance: 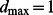 times and 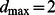 times the lattice spacing. Both choices lead to excellent agreement with the analytical solution for system GD. This demonstrates that the choice of the neighbourhood cut-off distance does not significantly affect the estimate for the entropy.

There is no exact result for the entropy of system KD. However, we notice from Fig. 1B that the conditional entropy is in good agreement with the Kikuchi approximation, a well established method for estimating entropies in lattice models. The conditional entropy can then be applied to the study of multi-component and/or multi-state lipid membranes on lattice [22], for instance to estimate the free energy of the system.

In continuous systems, the absence of the lattice prevents us from obtaining a rigorous expression for the Shannon entropy. However, we will see in the following that the conditional entropy can still be employed as a useful and accurate measure for disorder in continuous systems.

### Application to Brownian and Molecular Dynamics simulations: Lipid Mixing and Demixing

Multicomponent fluids and their mixing/demixing phenomena are an important field of present research, both in condensed matter physics [14] and for the understanding of biological systems [13]. In biophysics, especially important topics are phase separation and the formation of domains in mixed lipid bilayers [23], which are thought to play a crucial role in determining the function of membrane-bound proteins [11].

Here we show that Eq. (9) can be applied to quantify the level of demixing in multicomponent biomembranes, which are often modelled by means of computer simulations [24]. For this purpose, we use data from Brownian and molecular dynamics simulations and consider two different levels of coarse graining, in which each lipid is either represented by a single Lennard-Jones sphere (system CG-LJ), or by a finer level of detail represented by the MARTINI forcefield [25] (system CG-Mar).

**Figure 2:**
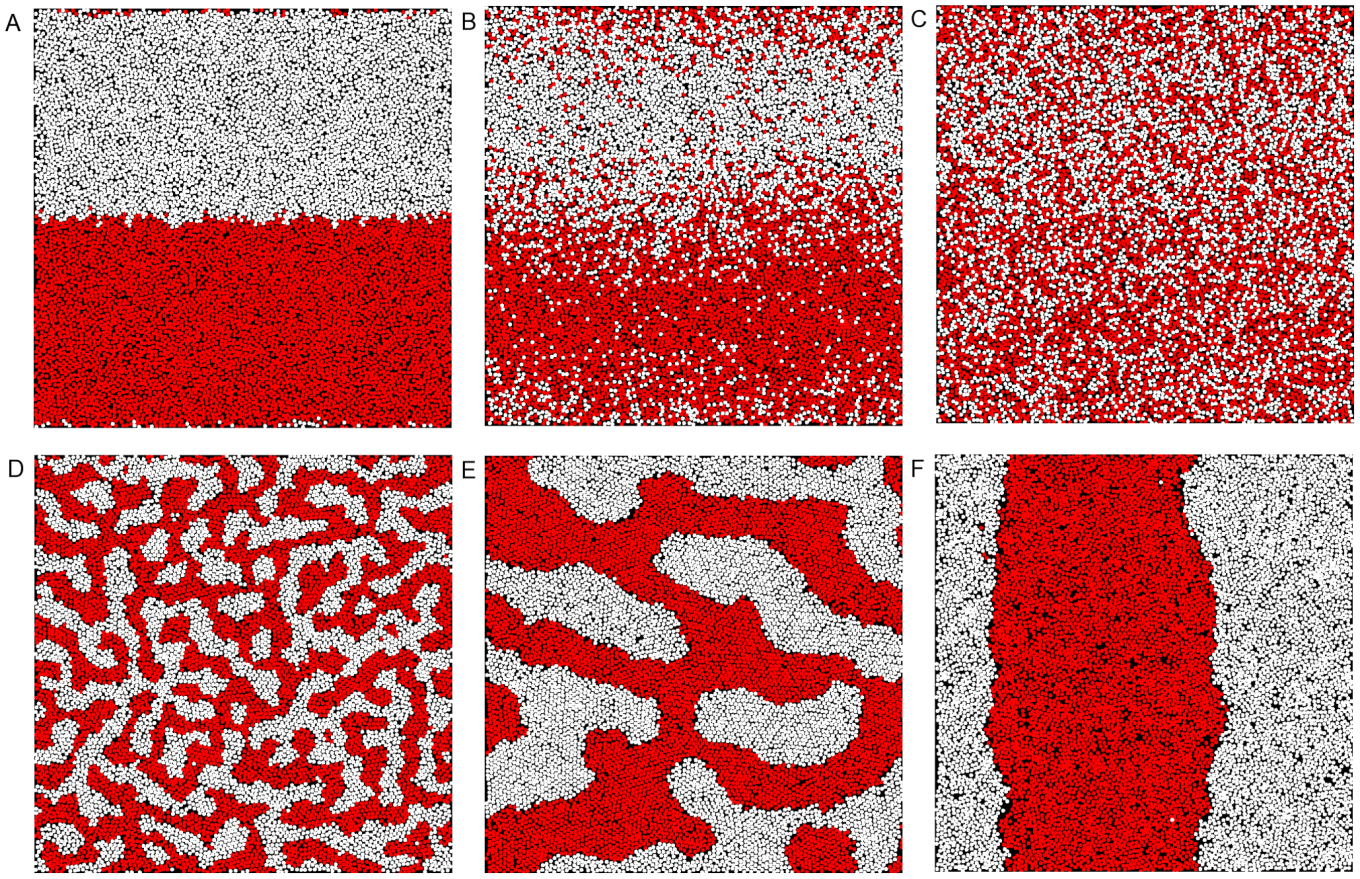
Brownian dynamics of mixing and demixing. Different snapshots of a coarse-grained Lennard-Jones (CG-LJ) binary fluid membrane of 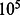 particles are shown. Mixing is followed from (A) at 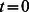, (B) at 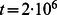 and (C) at 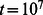; demixing takes place from (D) at 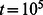, (E) at 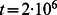 and (F) at 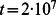.

Fig. 2 shows snapshots of a CG-LJ symmetric binary fluid of 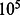 particles at different times. At 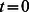, the two components A and B are well separated in two different regions of the box (Fig. 2A). Panels 2A–2C show how A and B gradually mix following simple diffusion (all the interactions between the particles are equivalent). Once the species are fully mixed, an attractive interaction between particles of the same type is switched on, leading to the formation of domains (2D), which then grow (2E), until the system has reached a fully demixed state (2F).

**Figure 3:**
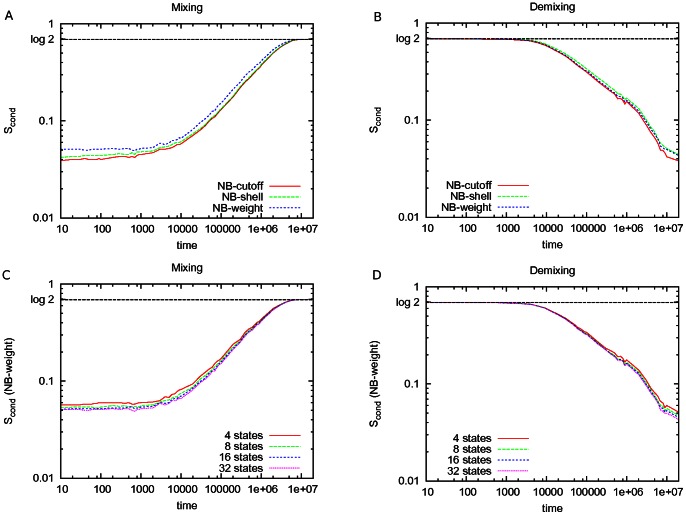
Conditional entropy during mixing and demixing. The conditional entropy is quantified during mixing (A,C) and demixing (B,D) of a CG-LJ binary fluid membrane of 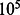 particles. In (A) and (B), different definitions of the particle neighbourhood are compared: NB-cutoff (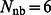), NB-shell (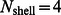, 2 shells), NB-weight (32 states) (see text). In (C) and (D), the conditional entropy is compared for different numbers of states of the neighbourhood after setting its definition to NB-weight.

**Figure 4:**
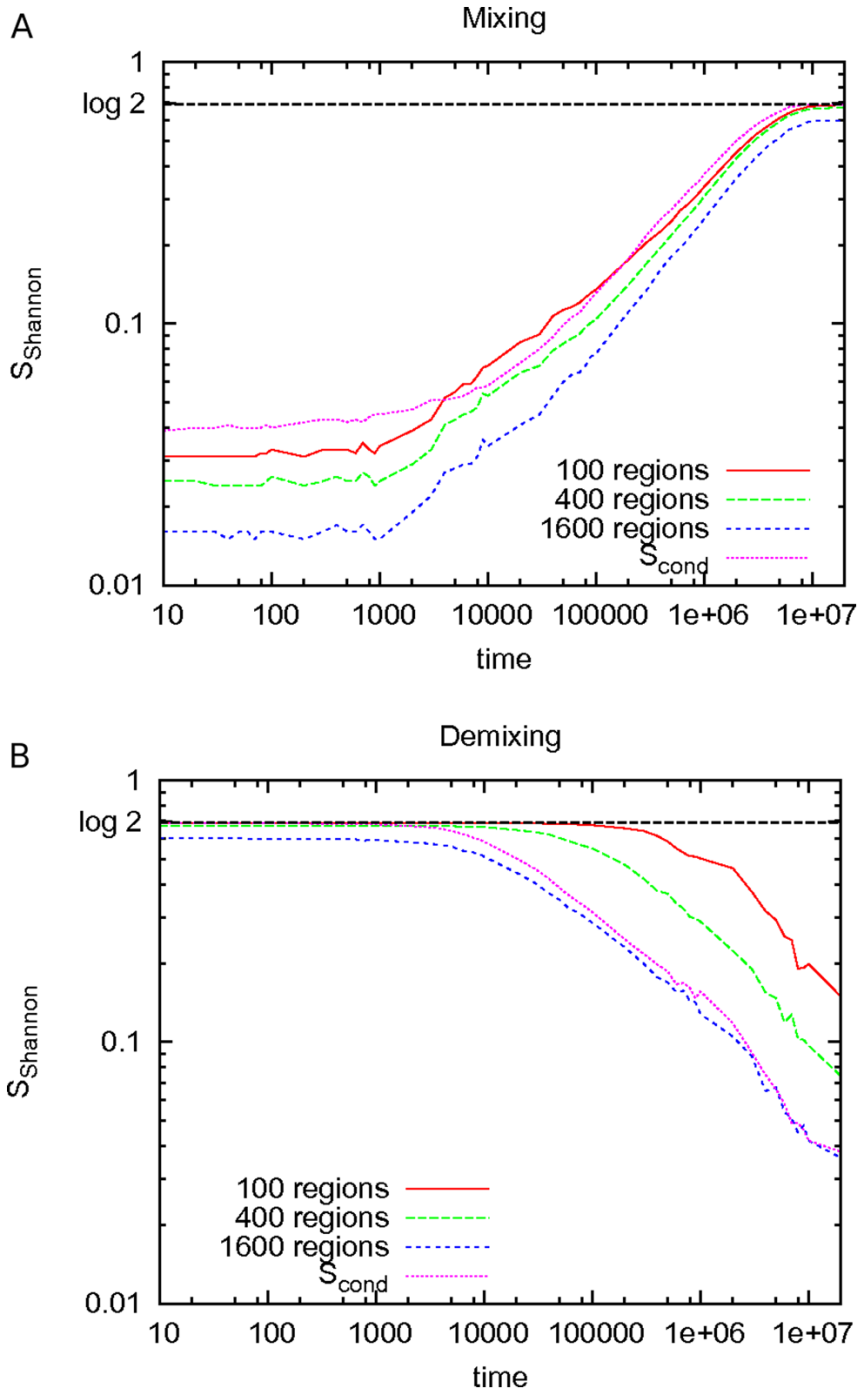
Shannon entropy during mixing and demixing. The graphs show the Shannon entropy as estimated from equation 4 during (A) fluid mixing and (B) fluid demixing. Data refer to the CG-LJ binary fluid membrane of 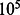 particles.

In order to estimate the disorder of the system via conditional entropy, we use Eq. (9). Here, the state of each particle 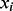 is simply its type (in Fig. 2 either A or B). The definition of the state of the neighbourhood depends on its size. Our choice was governed by the requirement to efficiently characterise, from single snapshots, the state of the local environment of each particle. However, if the number of possible states for the neighbourhood, 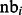, is too large, a single snapshot will provide insufficient sampling, and this can lead to an underestimation of the entropy. In the limit of a system of infinite size, 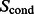 is zero when the two components of the fluid are separated, and 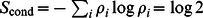 when they are perfectly mixed. These are also the limits for the thermodynamic entropy of an equilibrium system under the same conditions (note that, away from equilibrium, the term entropy only refers to a measure of disorder within the system). Fig. 3 shows the result of Eq. (9) applied on the CG-LJ binary mixture during mixing and demixing as a function of time, using three different definitions of 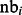. In Fig. 4, this result is compared to the “standard” Shannon entropy, as estimated by Eq. (4) [6].

To arrive at a suitable definition of the state of the neighbourhood, we first observe that the distance between particles is an appropriate quantity for ranking the relative importance of the neighbours. Then, to focus on local correlations, we can either use a cut-off distance, or a weight inversely proportional to the inter-particle distance. In order to avoid double-counting of the interactions, any particle 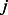 is counted as a neighbour of another particle 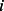 only if it is located in the upper half-space of the system from 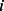, consistent with the definition used for the lattice case.

We now discuss the three different definitions of 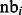 we used. In the first approach, we define the neighbourhood of each particle 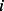 as the 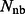 closest particles to 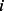. The state of the neighbourhood is then a vector of length 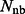 where component 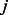 corresponds to the type of neighbour 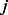:
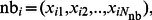
(11)


The number of possible states of the neighbourhood scales with the number of components in the mixture as 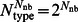. We refer to this definition as NB-cutoff in Fig. 3.

The second approach is similar but here, the neighbourhood is divided into shells containing a certain number of particles: the first shell contains the closest 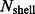 particles to 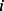, the second shell contains the second closest 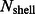 particles, etc. In this way, the state of the neighbourhood is defined as the number of particles of each type contained in each shell, a choice that reduces the number of possible states 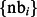. We refer to this definition as NB-shell in Fig. 3.

Finally, for the third approach, we associate to each particle 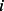 the following function:
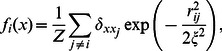
(12)where 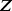 is a normalisation factor, 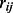 the distance between particles 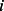 and 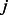, 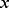 is the particle type, and 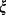 is a characteristic length scale of the system (which in our case is taken as half the average inter-particle distance). Physically, the quantity 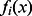 represents the frequency with which a particle of a certain type is observed in the neighbourhood of 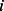 – where each neighbour is weighted with a factor decreasing exponentially with the distance. After appropriate binning, 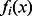 can be used as a discrete variable to label the states 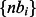 in Eq. (9). We refer to this definition as NB-weight in Fig. 3.

Interestingly, Fig. 3 shows that our quantification of disorder (Eq. 9) is robust with respect to the three quite different definitions of the state of the neighbourhood. All definitions lead essentially to the same value both during the mixing (3A) and demixing (3B) processes of our system, which is consistently bounded by zero and 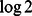. Fig. 3C and Fig. 3D show the convergence of 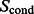 as we increase the number of states for NB-weight, a very desirable property of our quantification of disorder. In all of the considered implementations of Eq. 9, we chose the specific parameters so that the frequencies 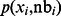 are estimated with sufficient sampling; for example by making sure that the conditional entropy reaches the maximum value in the fully demixed state.

Our method contrasts with previous approaches which use spatial binning of the system to quantify disorder via the Shannon entropy, and which strongly depend on the number of bins. In Fig. 4, our approach is compared to the evaluation of Shannon entropy according to Eq. (4), for which the system is divided into subregions of equal size [6]. Use of Eq. (4) exhibits a strong dependence on the number of selected subregions. This dependence is especially acute during the demixing process, which is of great relevance for the study of domain formation. The dependence is due to the loss of information within a length-scale smaller than the size of each subregion. This leads to a relative insensitivity of Eq. (4) to the onset and beginning stages of domain formation, so that subtle transitions towards demixing are not captured well. However, these partial transitions are expected to bear the highest biological relevance. For instance, upon division of the system into 100 regions each containing 100 particles, the entropy remains constant and equal to 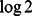 during the demixing process up to 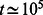, whereas the formation of local order is clearly observable from Fig. 2C and Fig. 2C. Choice of smaller subregions, however, will lead to convergence and sampling issues instead.

**Figure 5:**
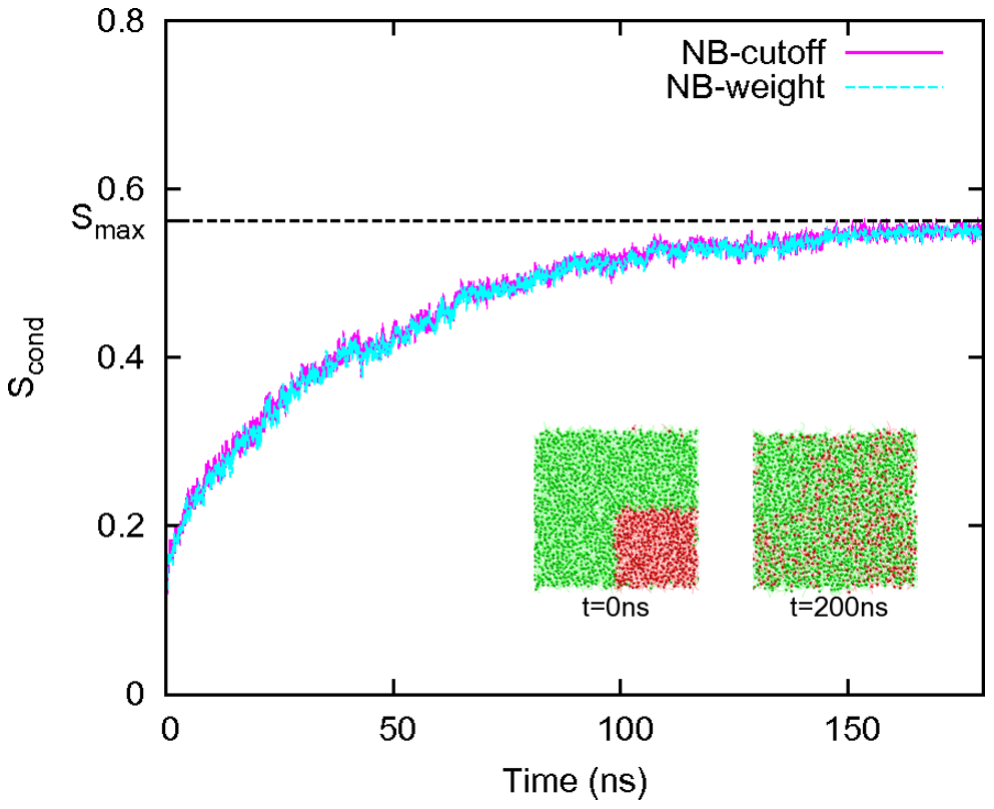
Lipid bilayer mixing. We show the conditional entropy quantification of mixing in a lipid bilayer, obtained using definitions NB-cutoff (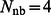) and NB-weight (8 states) for the state of the neighbourhood. The data were obtained from a coarse-grained molecular dynamics simulation of a biomembrane consisting of 504 POPC (red) and 1512 POPE (green) lipids with the MARTINI forcefield [25].

As we expect that one of the most important applications of our method could lie in the field of domain formation in biological systems, especially within lipid membranes, Fig. 5 shows an application of our method to a coarse-grained molecular dynamics simulation of a biomembrane. This membrane consists of a bilayer of 504 palmitoyl-oleoyl phosphatidylcholine (POPC) and 1512 palmitoyl-oleoyl phosphatidylethanolamine (POPE) lipids, based on the MARTINI force-field [25]. Fig. 5 shows the increase of conditional entropy (5d), estimated using NB-cutoff and NB-weight upon the mixing of lipids, from near zero at 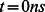 (5b) to 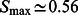 at 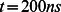 (5c). An implementation of the conditional entropy will be included in Membrainy, a tool-kit for lipid bilayers analysis available at http://code.google.com/p/membrainy/.

## Methods

Our Monte-Carlo simulations of the Ising model were carried out on a two-dimensional square lattice with periodic boundary conditions and a Hamiltonian 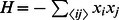, where the spins can take values 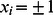 and the sum is performed over all of the nearest neighbours 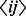. To generate an equilibrium ensemble of configurations, we used both Glauber and Kawasaki dynamics [26]. In the former, we attempt to change the state of a random spin at each step of a Monte-Carlo cycle according to the Metropolis algorithm. The final ensemble corresponds to the original system described by Onsager [2]. In Kawasaki dynamics, only the spins of two neighbouring sites are exchanged, so that the total magnetisation is conserved (we have chosen to work at zero magnetisation). This type of dynamics is suitable for studying thermodynamic quantities [22] and can be likened to diffusion processes [27] in lipid membranes.

We studied the mixing/demixing transition in a two-dimensional membrane by Brownian Dynamics (BD) simulations by using the software LAMMPS [28]. Specifically, we performed BD simulations of a symmetric Lennard-Jones (L-J) mixture of 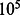 particles: 50% of type 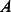 and 50% of type 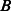. Each particle is spherical, and can be thought of as a lipid molecule within a lipid monolayer. The L-J potential governing inter-particle interactions is given by 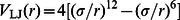, where 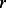 is the inter-particle distance and  the depth of the potential well; 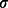 was set to one. The particles move inside a square box with periodic boundary conditions and size 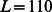. The overall density of the mixture is chosen such that the particles are closely packed but the overall system is fluid. A stochastic thermostat keeps the system temperature fixed at 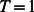. To study the mixing of the two components, we initially used identical particles that can only be distinguished by an assigned label(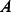 or 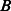), i.e., the interactions between all particles are identical. For each interaction (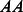, 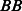 and 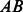), 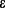 was set to 1 and a cut-off distance was used at 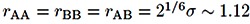, so that the force is repulsive only. To study demixing, we used the same cut-off for the 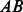 interaction (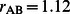), but the cut-off of the 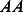 and 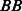 interactions was moved to 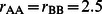 and 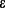 was set to 2. This choice introduces an attraction between particles of the same type, and enables observation of a demixing transition below the critical temperature 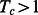.

Our method can also be applied to higher resolution molecular dynamics simulations of biomembranes [29]. To illustrate this, we have simulated a bilayer system consisting of 504 POPC and 1512 POPE lipids, explicitly solvated in 31191 water molecules. The bilayer was constructed such that, initially, all POPC lipids are clustered together in one corner of the bilayer (see Fig. 5) and mixing of the two lipid species was followed over 200 ns. The MARTINI model [25], which is used extensively for biomolecular simulations, was used to describe the lipids and surrounding solvent. This reflects a relatively high-resolution coarse-grained model that employs 4-to-1 mapping for heavy atoms. GROMACS 4.5 [30] was used for the MD simulations. The temperature was set to 323 K, well above the phase transition temperature of both lipids, and maintained by the Berendsen thermostat [31]. Semi-isotropic pressure coupling was used to maintain a pressure of 1 atm [31] to model an NpT ensemble. An integration timestep of 20 fs was used.

### Conclusions

In this paper, we have shown that the conditional entropy can be applied as a useful and accurate measure of disorder. Firstly, by considering an Ising model of spins on a lattice, we demonstrated that the conditional entropy represents a good approximation to the exact entropy of the system and the result is comparable to a well established method such as the Kikuchi approximation. Secondly, we applied our method to mixing and demixing in multicomponent fluid membranes. This is an important problem, with many implications for the biophysics of lipid biomembranes. At the same time, it also illustrates the usefulness of our approach, as quantifying the extent of demixing requires a careful evaluation of local correlations, which cannot be captured by standard mean field approximations.

It is important to note that rather than defining a new measure of non-equilibrium entropy, our main goal here is to provide an accurate quantification of disorder in multicomponent systems. Our approach based on conditional entropy has a number of desirable properties. First, it is, by construction, defined between the limiting values of the entropy of mixing corresponding to fully mixed and demixed states. Second, it is easy to implement, and, third, it is relatively insensitive to the details of the implementation (e.g. the definition of particle neighbourhood). The conditional entropy is a global measure of disorder which captures the local organisation of the system at small length-scales. This property makes our measure more accurate with respect to other global measures of disorder which are not generally able to capture local correlations (e.g., mean field approximation and Shannon entropy in Ref. [6]). Due to its generality, our approach can readily be applied to study correlations in system exhibiting any kind of disorder-order phase transition, such as fluid mixtures and liquid crystals.
